# Excellent Interim Treatment Response with Polatuzumab Vedotin

**DOI:** 10.4274/tjh.galenos.2020.2020.0042

**Published:** 2020-11-19

**Authors:** Özgür Mehtap, Gözde Dağlıöz Görür, Serkan Ünal, Ayfer Gedük

**Affiliations:** 1 Kocaeli University Facuty of Medicine, Department of Hematology, Kocaeli, Turkey; 2 Kocaeli University Facuty of Medicine, Department of Nuclear Medicine, Kocaeli, Turkey

**Keywords:** Polatuzumab, DLBCL, PPET-CT, Image

A 57-year-old male presented with cervical mass and B symptoms for the last 2 months. The patient had a history of stage II diffuse large B-cell lymphoma (DLBCL), with complete response after six cycles of rituximab-cyclophosphamide-doxorubicin-vincristine-prednisone (RCHOP) chemotherapy in 2010. At the time of diagnosis, his International Prognostic Index score was low-intermediate. Three years after RCHOP treatment, the patient suffered from a cervical mass again. Excisional lymph node biopsy confirmed relapse of DLBCL. After three cycles of rituximab-iphosphamide-carboplatin-etoposide (R-ICE) chemotherapy, the patient achieved a second complete response and underwent autologous stem cell transplantation. Five years after the transplantation, the disease relapsed again. Excisional lymph node biopsy revealed a relapse of the non-germinal type of DLBCL. The patient presented with the signs, symptoms, and laboratory findings of tumor lysis syndrome and paraneoplastic hypercalcemia. PET-CT imaging demonstrated widespread FDG uptake in the skeletal system (SUV_max_: 23.5), liver (SUV_max_: 16.2), mediastinal lymph nodes (SUV_max_: 7.4), abdominal lymph nodes (SUV_max_: 26.6), cervical lymph nodes (SUV_max_: 25.8), and left maxillary sinus (SUV_max_: 29.5) (Figure 1). The patient received six cycles of polatuzumab vedotin (1.8 mg/kg/day), rituximab (375 mg/m^2^/day), and bendamustine (90 mg/m^2^/day) every 21 days. After three cycles of polatuzumab-rituximab-bendamustine, the patient was evaluated again by PET-CT scan, which demonstrated the disappearance of all FDG uptake except that in the costal bones, which had decreased significantly (SUV_max_: 23.5 to 5.1) (Figure 2). This new drug can be effective and promising in this difficult-to-manage group of patients.

## Figures and Tables

**Figure 1:**
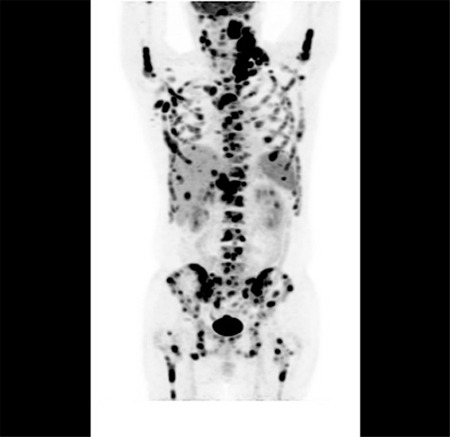
PET-CT imaging demonstrated widespread FDG uptake.

**Figure 2:**
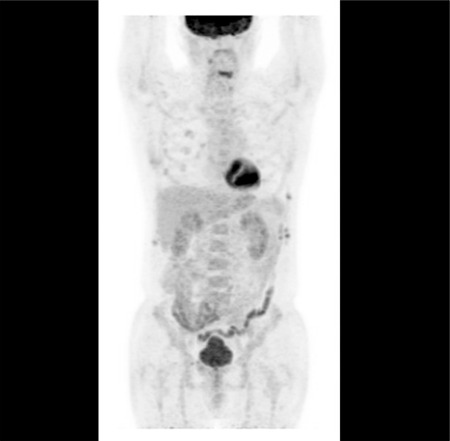
After three cycles of polatuzumab-rituximabbendamustine, PET-CT scanning demonstrated the disappearance of all FDG uptake except that in the costal bones.

